# The preferred retinal loci when the eyes converge

**DOI:** 10.1167/jov.24.9.15

**Published:** 2024-09-23

**Authors:** Norick R. Bowers, Josselin Gautier, Susana T. L. Chung, Martin S. Banks, Austin Roorda

**Affiliations:** 1 Herbert Wertheim School of Optometry & Vision Science, University of California, Berkeley, California, USA; 2 LTSI, Inserm UMR 1099, University of Rennes, France; 3 Herbert Wertheim School of Optometry & Vision Science, University of California, Berkeley, California, USA; 4 Herbert Wertheim School of Optometry & Vision Science, University of California, Berkeley, California, USA; 5 Herbert Wertheim School of Optometry & Vision Science, University of California, Berkeley, California, USA

**Keywords:** preferred retinal locus, binocular vision, fixation disparity, vergence eye movements, scanning laser ophthalmoscopy

## Abstract

The preferred retinal locus (PRL) is the position on the retina to which humans direct stimuli during fixation. In healthy normal eyes, it has been shown to be very stable across time and between different tasks. Previous measurements of the PRL have been made under monocular viewing conditions. The current study examines where the PRLs in the two eyes’ retinas are when subjects fixate binocularly and whether they shift when the demand for the eyes to converge is changed. Our apparatus allows us to see exactly where binocular stimuli fell on the two retinas during binocular fixation. Thus, our technique bypasses some of the issues involved in measuring binocular alignment with subjective techniques and previous objective techniques that use conventional eye trackers. These results show that PRLs shift slightly but systematically as the demand for convergence increases. The shifts cause under-convergence (also called exo fixation disparity) for near targets. They are not large enough to cause a break in binocular fusion. The fixation disparity we observed with increasing vergence demand is similar to fixation disparity observed in previous reports.

## Introduction

**Figure 1.**
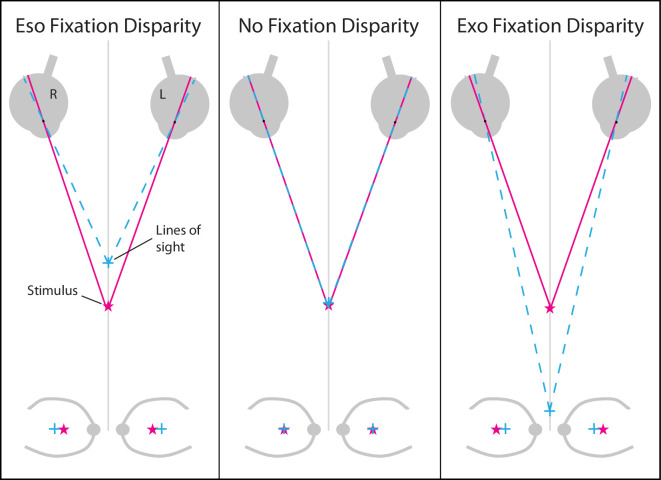
Schematic of eso and exo fixation disparity in the world and their respective projections on the retina. The panels from left to right illustrate, respectively, eso fixation disparity, no fixation disparity, and exo fixation disparity. In the upper panels the pink stars indicate the position of the stimulus in space, and the cyan crosses where the lines of sight from the two eyes intersect. The lower plots are schematic retinal images in conventional fundus-view orientation with the optic nerve and major blood vessels shown for reference. The fovea (cross) and the projection of the stimulus (star) are shown in each case. During eso fixation disparity (left), the eyes over-converge, causing the images of the stimulus to be shifted nasally (i.e., leftward in the left eye and rightward in the right eye). During exo fixation disparity (right), the eyes under-converge, which causes temporal shifting of the images of the stimulus.

As people look around the three-dimensional environment, they keep their two eyes aligned to maintain accurate perception of depth from stereopsis (Blakemore, 1970). They need to converge the eyes when fixating a near object and diverge the eyes when fixating a far one. But it is reported that people do not always fixate targets in depth accurately. They tend to fixate nearer than far targets (over-convergence, eso deviation) and farther than near targets (under-convergence, exo deviation) (Ogle, 1958; Ogle, Martens, & Dyer, 1967; London & Crelier, 2006; Fogt, 2023). This phenomenon is known as fixation disparity. Examples of eso and exo fixation disparities, and their effects on retinal images are shown in Figure 1.

**Figure 2.**
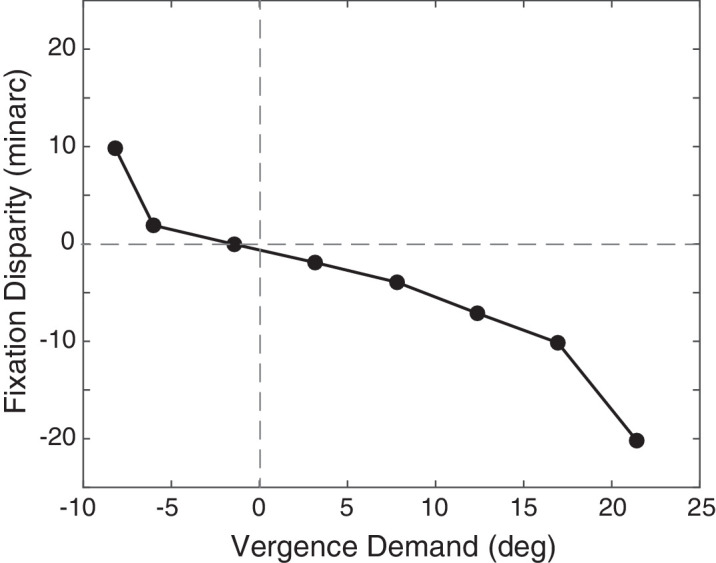
Fixation disparity measured subjectively using dichoptic nonius lines. Negative values of fixation disparity indicate exo disparity and positive values indicate eso disparity. Positive vergence demand indicates convergence. Fixation disparity in minutes of arc is plotted as a function of vergence demand in degrees. Subjects maintain good alignment for a range of vergence demands before fixation disparity becomes large. Adapted from (Ogle et al., 1967).

Fixation disparity has been measured both subjectively and objectively. In subjective measurements, subjects are told to maintain accurate fixation on a binocular target while adjusting the horizontal positions of dichoptic vertical lines to make them aligned perceptually (Ogle, Martens, & Dyer, 1967; Duwaer & Van Den Brink, 1981; Jaschinski, Kloke, Jainta, & Buchholz, 2005). Different vergence demands are created by placing prisms in front of the eyes or by varying the distance of the binocular fixation target. The assumption behind the technique is that when subjects report that the dichoptic lines appear to be aligned, the two lines are stimulating pairs of corresponding retinal points in the two eyes. An example of results from a subjective experiment is shown in Figure 2. From left to right, the vergence demand proceeds from divergence to convergence. The fixation disparity is plotted on the vertical axis in minutes of arc: negative values for exo disparity (under-converged) and positive ones for eso disparity (over-converged).

According to this figure, the disparity remains near zero for a range of vergence demands, but different individuals exhibit different ranges of vergence demands for which they can keep their eyes aligned. Clinical experience shows that demands for which the patient exhibits significant fixation disparity cause eye strain (Sheedy & Saladin, 1978; Jaschinski, 2002). For example, fixation disparity is a better predictor of eye strain than phoria (Yekta & Pickwell, 1986). Accordingly, these measurements are used to prescribe spectacle corrections to reduce such strain (Scheiman & Wick, 2008).

The amplitude of measured fixation disparity varies across studies and techniques. For example, subjective techniques sometimes find smaller fixation disparities than objective techniques (Fogt & Jones, 1998a; Jaschinski, 2018). The difference between disparities measured objectively and subjectively has been attributed to shifts in corresponding retinal points (Fogt & Jones, 1998a; Fogt & Jones, 1998b; Brautaset & Jennings, 2006; Jaschinski, 2018). For example, when a stimulus requiring large convergence is presented, the eyes attempt to make the appropriate vergence movement, but are not quite able to do so. To make up for the residual oculomotor error, corresponding retinal points are said to shift thereby helping to maintain perceived alignment. Said another way, the idea is that subjects will not always fixate using the same retinal locus for fixation, but instead may adopt slightly eccentric fixations in the two eyes to stimulate the shifted corresponding points. The evidence for such shifting of corresponding points is decidedly mixed (Hillis & Banks, 2001).

The idea behind measuring fixation disparity is to determine the vergence demands for which the patient can fixate a binocular target such that the images of the target fall on the foveal centers in both eyes. There are three issues here: 1) whether the subject actually uses the centers of the foveas for fixation, 2) whether the optical projection from an external point to the retina remains the same for all vergence demands, and 3) in objective measurements, whether a given eye tracker measures the directions of the lines of sight correctly. We next expand on these three issues.

Consider first the issue of whether the centers of the foveas are used for binocular fixation. During careful fixation of a monocular target, most subjects do not place the image of the stimulus on the exact foveal center. Instead they use a nearby location that is on average approximately 5 minarc from the position of peak cone density (Putnam et al., 2005; Wilk et al., 2017; Bowers, Boehm, & Roorda, 2019; Wang et al., 2019; Reiniger, Domdei, Holz, & Harmening, 2021). This retinal position is called the preferred retinal locus (PRL). The PRL is quite stable within individuals. It does not change significantly over time (Kilpeläinen, Putnam, Ratnam, & Roorda, 2021; Reiniger et al., 2021) or across tasks (Bowers, Gautier, Lin, & Roorda, 2021). It is not known how the PRLs in the two eyes are positioned relative to each other and whether those PRLs change with vergence. The fact that objective and subjective measurements of fixation disparity yield somewhat different results could be explained by shifting PRLs under different vergence demands.

The second issue concerns the projection from an external point to an image point on the retina. If we know the positions of the eyes' nodal points, we can map positions of points in space into retinal coordinates. The eye has a primary and a secondary nodal point. A ray passing through the primary nodal point exits the secondary point at the same angle relative to the optical axis. Tracing these rays yields an accurate mapping from object points into retinal coordinates. In fixation-disparity experiments the stimuli, whether binocular or dichoptic, are defined in head coordinates. To convert from these coordinates into retinal coordinates, we need to know the head-centered coordinates of the primary and secondary nodal points and the head-centered coordinates of retinal surface points. Unfortunately, nodal-point positions in head-centered coordinates change with eye rotation and accommodation. With eye rotation, the nodal points translate relative to the head because the nodal points are in front of the eyes' centers of rotation: they translate nasally with convergence. By changing the head-centered positions of the nodal points, eye rotations change the ray tracing from object points to retinal points. Given that conventional eye trackers must infer positions on the retina from measurements on anterior parts of the eye, this effect must be eliminated or taken into account to determine the retinal positions of corresponding points from fixation disparity data. With accommodation, the positions of the nodal points shift toward and away from the cornea, and this has a small effect on the ray tracing from object points to retinal coordinates, which might also need to be taken into account. These optical effects are usually not accounted for in measurements of fixation disparity, whether done subjectively or objectively.

The third issue concerns the precision and accuracy of the binocular eye trackers themselves. Most video-based trackers lack the requisite resolution to track gaze position on a fine enough scale to measure changes in fixation disparity (Kimmel, Mammo, & Newsome, 2012; Holmqvist & Blignaut, 2020; Niehorster, Zemblys, & Holmqvist, 2021). Furthermore, these eye trackers are prone to artifacts caused by changes in pupil size (Choe, Blake, & Lee, 2016; Hooge, Holmqvist, & Nyström, 2016; Nyström, Hooge, & Andersson, 2016). The pupil constricts with convergence and dilates with divergence (Eadie & Carlin, 1995), which may cause vergence-related bias in tracking. Although some studies have attempted to correct these artifacts (Jaschinski, 2016; Jaschinski, 2017), the corrections are not always used. Furthermore, one would have to carefully control for anything that could affect pupil size between calibration and testing (i.e., stimulus luminance, monocular vs binocular calibration, subject arousal, etc.) to ensure there are no systematic measurement errors caused by changes in pupil size. Measurements taken with video eye trackers also tend to be more variable than those taken with higher-resolution trackers, such as the Dual-Purkinje Image eye tracker and scleral search coils (Kertesz & Lee, 1987; Erkelens, Steinman, & Collewijn, 1989; Fogt & Jones, 1998a; Brautaset & Jennings, 2006). The current study has the significant advantage of avoiding the need to calibrate for any pupil-size artifacts by relying on direct imaging of the stimulus on the retina. In addition, most eye trackers rely on the use of calibration techniques that may impose different demands on the subject than the experiment itself, such as monocular calibrations being done for each eye independently.

In summary, previous measurements of fixation disparity may be inaccurate. The goal of the current study is to determine where on the two retinas a binocular stimulus falls when the observer fixates the stimulus and the vergence demand changes. We present new data in which we image the retinas and the stimulus falling on the retinas simultaneously as different vergence demands are presented. The data will determine, once and for all, the actual fixation disparity for different vergence demands.

## Methods

### Human subjects

Five subjects participated in the experiment. They had minimal or no refractive error and self-reported to have no visual or neurological disorders. Experimental protocols adhered to the conditions set by the institutional review board of the University of California, Berkeley. Eye dominance was determined for each subject using a conventional method that involved determining which of the eyes was chosen to view a distant target through an occluding aperture made with the hands held at arm’s length (https://www.aao.org/eye-health/anatomy/eye-dominance). Four standard clinical measures were made to assess the binocular function of our subjects. First, the presence of tropia was assessed using the unilateral cover test. Second, the phorias at distance (4 m) and near (40 cm) were measured using the alternating cover test and employing a calibrated prism bar to negate the eye motion. Subjects wore distance or near correction, if necessary, to make the near and/or distant targets clear. Third, any indication of either left- or right-eye suppression was assessed using the Worth 4-dot test at distance (4 m) and near (33 cm) (Roper-Hall, 2004). Finally, we tested for stereoacuity of 250 seconds of arc or better using the Forms component of the Randot stereotest (Stereo Optical, Chicago, IL) at the prescribed viewing distance at 40 cm, with correction if needed.

### Apparatus

Data were collected using a binocular tracking scanning laser ophthalmoscope (TSLO). A binocular version of such an ophthalmoscope can use a single platform, splitting the light between the left and the right eye (Stevenson, Sheehy, & Roorda, 2016; Hofmann, Domdei, Jainta, & Harmening, 2022), but the system used for this study consists of two independent TSLO systems (Sheehy et al., 2012) that are temporally synced. Each TSLO has the capability to image and track the retina, and to display stimuli on the retina directly. For imaging, an 840-nm near-infrared point source is swept across the retina in a raster pattern. This is enabled by two scanners, a 16-kHz horizontal resonant scanner and a 30 Hz vertical galvanometer scanner. The light scattered from the retina is descanned through the optical path, passed through a confocal pinhole and detected by a photomultiplier tube. Images of the retina are constructed pixel by pixel over time by combining the detected light intensity with positional information read from the mirror scanners. For this experiment, the size of the raster was 6 × 6° digitized into 512 × 512 pixels, yielding a pixel resolution of approximately 40 arcsec. The images obtained by each TSLO were combined to create two temporally synced movies of the retina at a frame rate of 30 Hz. From the subject’s perspective the scanning TSLO rasters appear, when fused, as a single red display with an effective luminance of just over 1 cd/m^2^ (Domdei et al., 2018). Stimuli can be effectively “drawn” directly into the raster display by turning off the laser during specific points of the scan to create black-on-red decrement stimuli. Figure 4 shows a schematic of the system.

**Figure 3.**
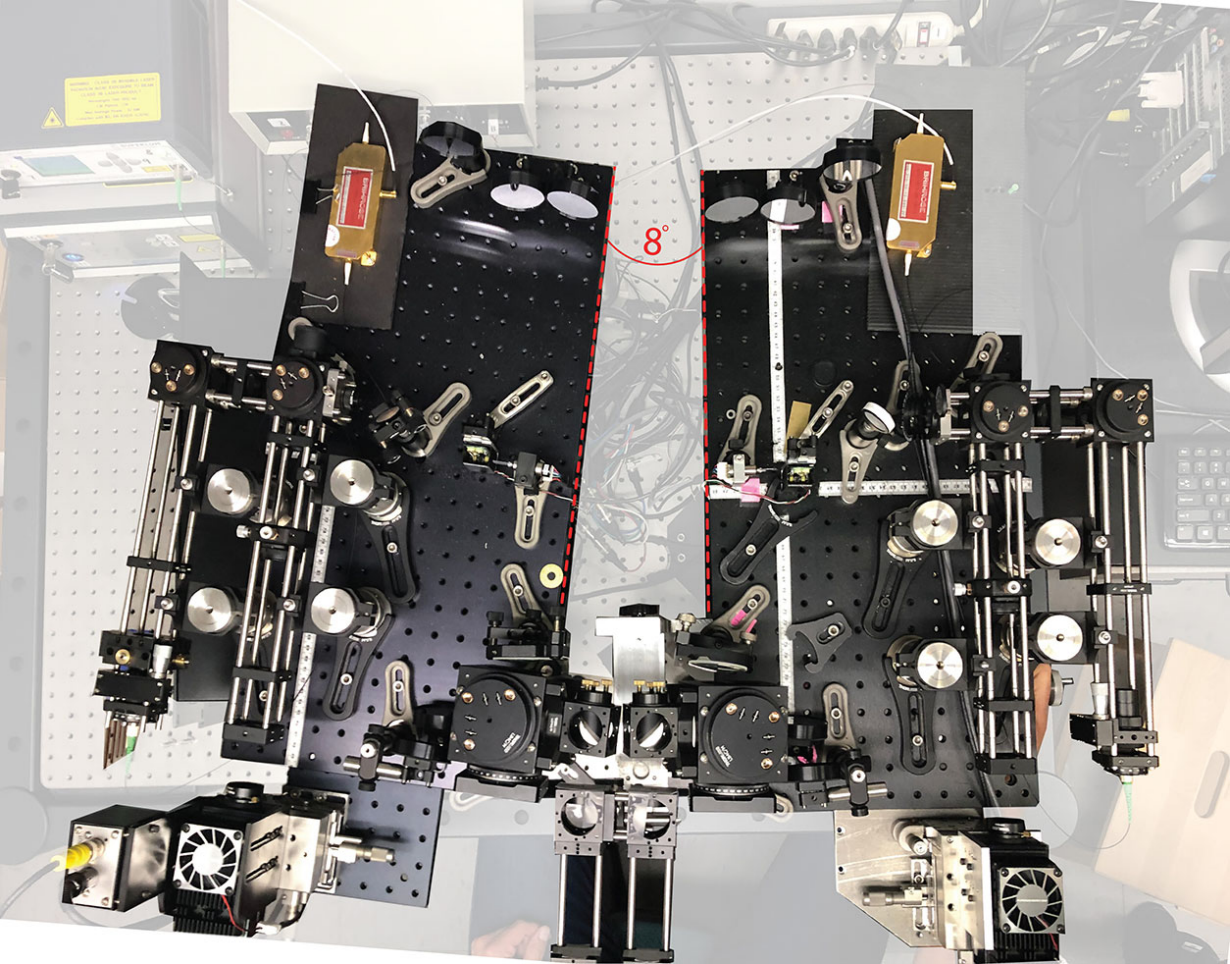
The Binocular TSLO with an 8° vergence demand. The red, dashed lines indicate the angle of rotation.

**Figure 4.**
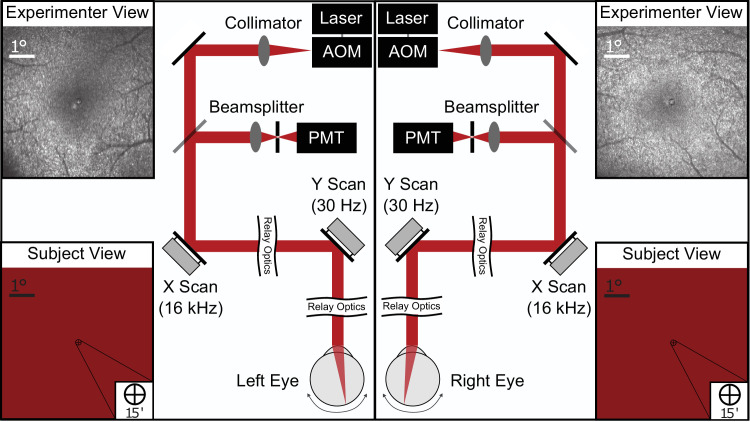
Schematic of the binocular TSLO. Two separate TSLO systems are temporally synced to present images to the two eyes independently. In both cases, a point source of light from the 840nm superluminescent diode is routed through the acousto-optic modulator and sent to the subject’s retina after reflecting off mirrors attached to the two scanners (fast horizontal scan and slow vertical scan). The reflected light is descanned through the optical path and sent to the PMT for imaging. The laser can be turned off at specific points in the raster scan to project a decrement stimulus onto the retinal surface directly. Along the sides of each schematic are examples of the experimenter’s view of the subject’s retina (top) and the subject’s view of the raster and decrement stimuli (bottom). All retinal images are in fundus view. Note that during the experiment the two images of the raster were fused (i.e., seen as a single display). Subjects did not report diplopia during the experiment, but this was not explicitly measured.

To facilitate binocular alignment, each TSLO was mounted on its own optical breadboard. The left TSLO was placed on a larger optical table and the right TSLO was mounted on an X-Y-Z adjustable instrument stage (Figure 3). Alignment of the subject in the system was a two-stage process. The subject positioned themselves in the system with a chinrest and temple pads. The temple pads helped to prevent any rotation of the subject’s head. The X-Y-Z position of the chinrest stage was adjusted until a clear and sharp image was obtained from the subject’s left eye. Then the entire right TSLO system was adjusted in X-Y-Z to optimize the image of the right eye.

To enable accurate setting of the scanned field size, the raster orientation, and the vergence demand, we used a custom-built binocular model eye. The eyes were assembled on a single cage hardware system (Thorlabs, Newton, NJ) to ensure that the optical axes of the two eyes were parallel to each other. Each eye used a 125-mm focal length lens with a 4-mm aperture (pupil) centered on each lens. The model eye’s retina was a printed square grid (black lines on white paper) that was placed at the secondary focal point of each lens. Each square of the grid subtended an angle of 0.5°. The printed grid had a small dot at one crossing point that was carefully centered with the optical axis of the lens and the cage system. The grid lines were aligned to be parallel and perpendicular with the cage system. To establish the 0° vergence condition, the two TSLOs were adjusted to center the scanning beams on the left- and right-eye pupils of the model eye and the direction of each beam was adjusted to ensure that 1) the grid in the scanned image was aligned with the raster (i.e., the raster scans were parallel and perpendicular) and 2) the center of the grid was in the center of each image. The exact field size was established by setting the angles of the scanners until a 6 × 6° field was visible on each eye.

The vergence demands were created by rotating the entire left TSLO system. The exact angle was set by measuring a lateral shift in the model eye images by the appropriate amounts (4° and 8°). The same binocular alignment procedure described above was used after setting each vergence demand. A photo of the binocular TSLO with the left system rotated for an 8° vergence demand is shown in Figure 3.

### Experimental protocol

Subjects were given a simple task to keep them fixating accurately. The stimuli were either a ⊕ or ⊗ symbol and would randomly switch every 2 to 4 seconds. Subjects were instructed to fixate the stimuli throughout the trial and indicate with a key press whenever the symbol switched. Examples of the stimuli in the subject’s and experimenter’s views are shown in Figure 4. The stimuli were approximately 15 minarc in size. There were four experimental conditions: monocular (for each eye), binocular with 0° vergence demand, binocular with 4° demand, and binocular with 8° demand. Those demands correspond, respectively, to 0, 7, and 14 prism diopters. In the monocular condition, one eye fixated the target and videos were recorded of that eye while the other eye was patched. Although the unmeasured eye was not stimulated in the monocular condition, the vergence demand of the system was set to 0°. In the binocular conditions, subjects fixated with both eyes and videos of both eyes were recorded. The vergence demands were 0° (i.e., parallel gaze), 4° (86-cm viewing distance), and 8° (43-cm viewing distance). Three 15-second movies were recorded from each condition. Conditions were counterbalanced between the subjects. Data were recorded using a natural pupil (no cycloplegia) with the lights off. This ensured the pupil was sufficiently large to enable high-fidelity imaging.

### Eye movement analysis

Eye movement traces were computed directly from the recorded TSLO videos. Because this system uses a raster-scanning technique that generates an image of the retina over time, the constant eye movements give rise to unique distortions within each recorded frame which encode eye motion information at rates greater than the frame rate (Mulligan, 1997). Our general approach to recover the eye motion is described in previous publications (Stevenson & Roorda, 2005; Stevenson, Roorda, & Kumar, 2010). The software package we used to implement it is called ReVAS (Agaoglu, Sit, Wan, & Chung, 2018). The basic approach is as follows. First, a coarse reference frame is generated by aligning every frame of the movie using cross-correlation. Then, each of the aligned frames are broken down into horizontal strips and shifted, based again on a cross-correlation, to align with the coarse reference frame. The fine reference frame is made by averaging all the aligned strips from all the frames. In this way, a high signal-to-noise ratio, relatively distortion-free reference frame is generated. Once this high-resolution reference frame is obtained for each video, the frames of the original video are again broken down into horizontal strips and cross-correlated against this fine reference frame to obtain an eye trace from the movie. The temporal resolution of this trace is determined by the number of strips used in each movie; in the current study. 16 strips were used per movie to acquire an eye trace at 480 Hz. The spatial sampling resolution of the eye trace is determined by the size of a pixel of the retinal image, which is approximately 40 arcsec.

The tracking depends on image quality. Of course, no tracking was possible during blinks. Whenever there were disruptions in the tear film, or if the eye shifted its position too much relative to the scanning beam, quality would degrade and tracking would fail for those frames of the video. Tracking failures manifest as high-amplitude white noise in the traces. Such eye traces were identified manually and excluded from further analysis.

Finally, if the eye makes torsional eye movements (rotations that are roughly around the line of sight) or the subject rotates their head, and consequently their eyes during the measurement, they manifest as a sawtooth waveform in the eye motion traces at exactly the frame rate. This artifact adds noise to the gaze position, but does not affect the mean gaze direction so we did not remove it.

### Eye movement alignment and PRL identification

As described elsewhere in this article, the eye motion traces for each condition in the experiment were computed using a different reference frame. As such, comparison of the stimulus positions on the retina from one condition to the next are arbitrary unless all traces are put into one coordinate system. The following steps were taken to register all data into a single coordinate system for each eye and each subject.

First, the exact X-Y trajectory of the fixated stimulus on the retina was computed for each condition by adding an offset to each eye motion trace to anchor it to the fixated stimulus. This process was relatively straightforward because the stimulus is encoded directly into the TSLO video. At this stage the trajectory of the stimulus for each movie is still relative to its own reference frame.

Second, the reference frame for each movie, along with its corresponding X-Y trajectory of the fixated stimulus, was aligned to a single master reference image for each eye. For all subjects, the fine reference frame that was made for the 0° vergence condition was chosen as the master reference. These alignments were accomplished by using cross-correlation. Whenever cross-correlation could not be used (usually owing to changes in retinal reflectance), the reference frame was aligned manually to the master reference. This adjustment was necessary for 5 of the 30 total alignments. Because any incorrect registration of the reference frames onto the master reference would yield erroneous offsets of the eye trace in the retinal image, these reference frame alignments were verified by systematically offsetting each reference from the master in one-pixel steps until the cross correlation of the two images indicated an offset in the opposite direction. For most subjects this gave us a verification for the registration of ∼1–3 pixels.

After the X-Y offsets needed to align one condition’s reference to the master were found, each reference frame was rotated and cross-correlated against the master reference again in a series of 0.1° steps and the highest peak correlation was used to obtain a measure of torsional differences between a reference and the master. These alignments were verified manually by ensuring that the position of the stimuli on the retina from the original movie matched both the reference frame for each condition, as well as the master.

**Figure 5.**
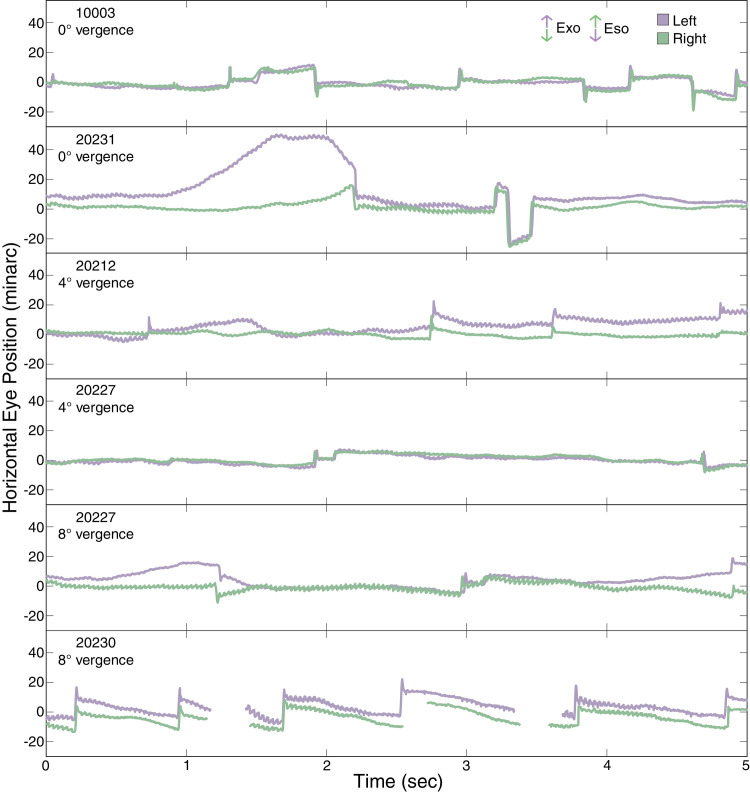
Binocular eye traces. Examples of eye motion for the left (purple) and right (green) eyes from all subjects and all conditions. Horizontal eye position in minutes of arc is plotted across time in seconds. Vergence demands for each example are indicated. Each eye trace is plotted relative to the PRL identified in the monocular condition: that is, 0 represents the monocular PRL horizontal location.

Once all traces were aligned to a master reference frame, the PRL was determined for each condition as the peak of the bivariate kernel density estimation (Botev, Grotowski, & Kroese, 2010) of the fixated stimuli positions across the retina. We chose this method to compute the PRL because it is not prone to shifts caused by occasional losses of fixation by one eye like those shown in Figure 5. When we computed the PRL as either the mean or median of fixation location (data not shown), the magnitudes of fixation disparity were about twice as large. To quantify the precision and robustness of each PRL estimated from the peak density, we computed 400 additional PRLs computed from 200 randomly drawn samples of the eye motion trace. The estimates formed a tight cluster of points around the full PRL and the interquartile ranges of each cluster are plotted as error bars in Figures 8 and 10.

**Figure 6.**
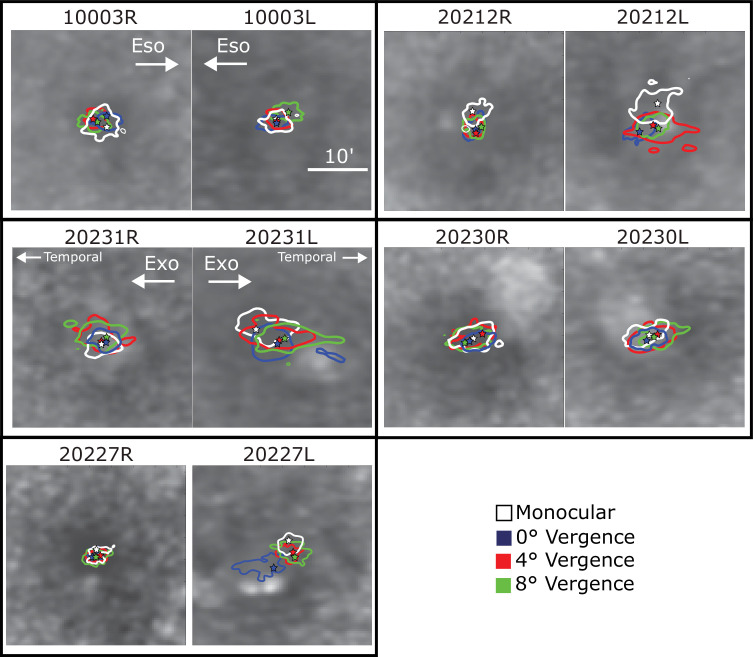
Isoline contours for each subject and vergence demand. The contours that enclose the central 68% of the fixated stimulus locations are plotted on the associated retinal images. Different panels show the data for different subjects and demands. White represents the contours from the monocular condition. Blue, red, and green represent, respectively, the contours for the 0°, 4°, and 8° vergence demands in the binocular condition. The central stars represent the peaks of the kernel density functions; this is a measure of the PRL.

**Figure 7.**
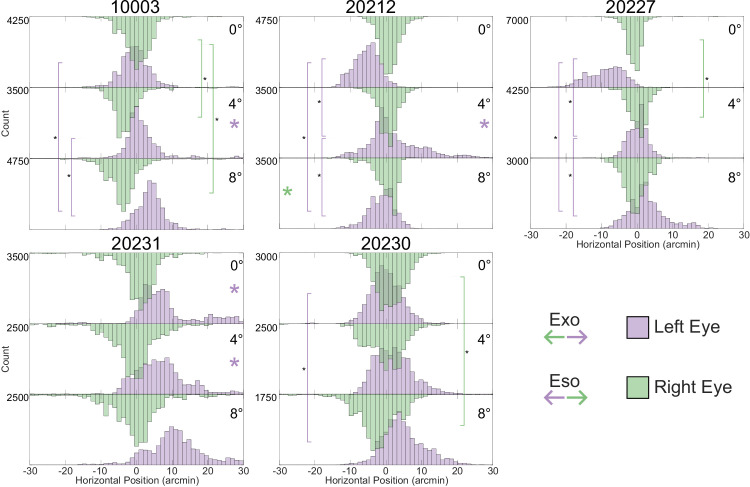
Horizontal eye positions in left and right eyes. The upward (purple) histograms are for the left eye and the downward (green) histograms are for the right eye. Shifts consistent with exo and eso fixation disparity directions are indicated in the legend. The 0 point in each histogram represents the monocular PRL for each subject. The central tendency for the distributions shift in such a way that the subject would be increasingly underconverging on the target as the vergence demand increases (exo fixation disparity). This trend can be difficult to discern from these figures, but is discussed in more detail below. The small colored asterisks on the histograms indicate distributions that had high skewness (>0.5). The asterisk is on the right for positive skewness and on the left for negative. The vertical stems indicate statistically significant changes. Significance was tested by a sign-rank test with a post-hoc Bonferonni correction, wherein 100 samples were taken from each distribution and tested against each other in a pairwise fashion for each subject. This was done 1,000 times to obtain 1,000 *p* values. Significance is only flagged if the entire range of the 95% confidence interval from these 1,000 *p* values fall below the significance threshold. There were generally more significant differences in the left (nondominant) eye compared to the right.

**Figure 8.**
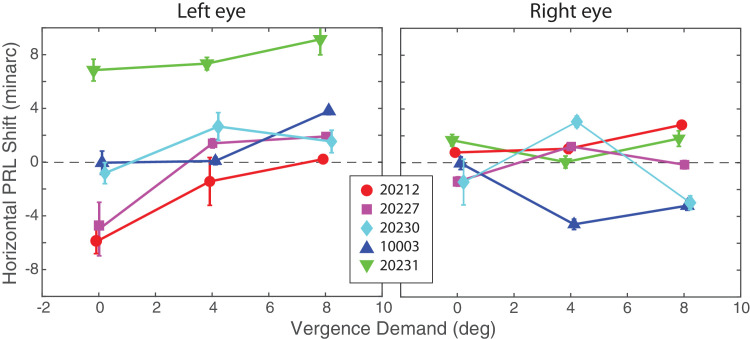
Horizontal PRL shifts in left and right eyes. Horizontal position of the PRL under binocular viewing is plotted for different vergence demands relative to the position under monocular viewing. Left panel shows the shifts in the left eye and right panel shows them in the right eye. Positive values correspond to rightward shifts in the fundus view. The data from each of the five subjects is plotted separately (see legend). Error bars represent the horizontal interquartile range of each PRL estimate (25th–75th percentile). Each distribution was bootstrapped with 1% of the original data, 400 times. Over each new distribution composed of 400 bootstrapped PRLs, the IQR was computed along each horizontal marginal.

**Figure 9.**
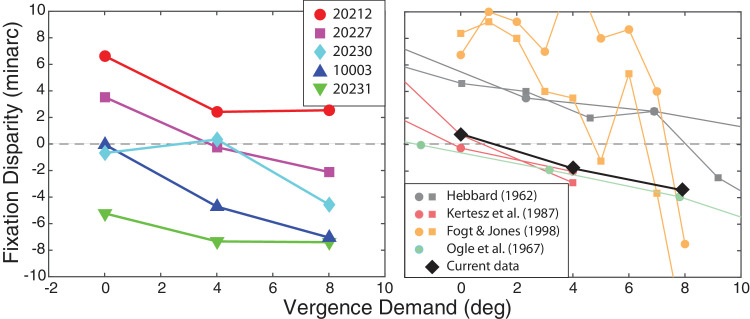
Fixation disparity. (Left) Mean fixation disparity for each subject in the current study. Fixation disparity in minutes of arc is plotted as a function of vergence demand in degrees. Data from each subject are plotted with different colors and symbols (see legend). We defined fixation disparity for each vergence demand as the difference between the horizontal PRLs in the two eyes, identified at the peak of the kernel density estimation, relative to the monocular PRLs. Positive values indicate an eso fixation disparity (over-convergence) while negative values indicate an exo fixation disparity (under-convergence). (Right) Mean fixation disparity from our experiment compared to the mean fixation disparity from previous experiments. Fixation disparity is plotted as a function of vergence demand. Data from each study is plotted in a different color (see legend). Our data averaged across subjects are represented by black diamonds. Data from the other studies have been averaged across subjects. Circles represent data obtained with subjective methods and squares data obtained with objective methods. The Hebbard (1962) data are from one subject, the Kertesz (1987) data from four, the Fogt (1998a) data from four, and the Ogle (1958) data from one.

**Figure 10.**
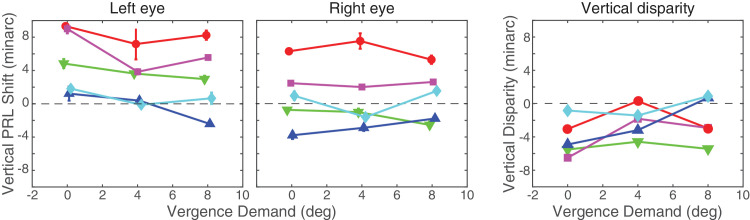
Vertical PRL shifts. (Left and center) Mean vertical PRL shifts relative to PRLs with monocular viewing. The left panel shows the data for the left eyes and the center panel those from the right eyes. Each color represents the data from one of our subjects as indicated in Figure 9. Vergence demand is the stimulus for horizontal vergence. (Right) The vertical disparity resulting from the left- and right-eye shifts. The error bars represent the vertical interquartile range of the PRL estimates (see Figure 8).

The isoline contour was computed from these same densities by finding the contour area whose cumulative probability is equal to 68%. Hence this contour encompasses approximately the central 68% of the fixated stimuli positions across the retina (Castet & Crossland, 2012). The isoline contours for each condition on the master reference image are shown in Figure 6. The area within the isoline contour is the isoline area or (ISOA). This technique is described in detail in Bowers et al. (2021).

We used a sign-rank test to determine if and when the horizontal position of the eye differed between the conditions. Specifically, we took 100 random samples from each of the conditions and tested them against each other in a pairwise fashion. This was done 1000 times and significance was only assumed if the entire range of the 95% confidence interval of the resulting 1000 *p*-values fell below the significance threshold.

## Results

We measured the retinal positions of the fixation target as observers fixated with one eye or with both eyes under different vergence demands. Our goal was to determine if PRLs shift as the eyes converge to targets at different vergence distances. Table 1 lists all clinical measures of binocular vision. Those results rule out any binocular vision dysfunction in our subjects. All subjects showed right-eye dominance. No tropias were detected using the unilateral cover tests. The phorias, measured in prism diopters, were all within normal limits with slightly more exophoria for near, as expected (Tait, 1951). No subjects exhibited evidence of left- or right-eye suppression at near or far on the Worth four-dot test. All subjects had stereo of at least 250 seconds of arc using the Randot stereotest, implying that subjects were unlikely to have suppression in one eye, or a microtropia. The Worth four-dot test was not performed on subject 20227, but that subject was found to exhibit no left- or right-eye suppression on the Randot suppression test.

**Table 1.** Subject data. Units are in prism diopters. Check marks indicate no binocular dysfunction was detected on the task. NM, not measured; XP, exophoria.

			Phoria		Worth 4-dot		
Subject ID	Age	Dominant eye	Distance	Near	Distance	Near	Randot
10003	55	right	Ortho	1 XP	✓	✓	✓
20230	26	right	2 XP	2 XP	✓	✓	✓
20231	31	right	2 XP	6 XP	✓	✓	✓
20212	26	right	Ortho	4 XP	✓	✓	✓
20227	28	right	Ortho	Ortho	NM	NM	✓

Figure 5 shows representative examples of the horizontal gaze directions for the left and right eyes for each binocular convergence demand during the course of a trial. All the traces that were used in this study are included as Supplemental Material. Both eyes remained relatively well-aligned throughout the course of a trial, but one eye would occasionally diverge. Examples of this can be seen in the complete traces for subjects 20231, 20212, and 20227 at 8° vergence. The most frequent and largest deviations occurred for subject 20231 and were especially prevalent for the 8° vergence condition. Subject 20231 also had the largest phorias at distance and near (Table 1), but overall there were no systematic trends observed. When such deviations did occur, it was often due to greater movement of the nondominant (left) eye. This is evidenced by a greater tendency toward positive skewness in the distribution of the left-eye traces (average skewness across all subjects and conditions = 0.27) compared with the right eye (average skewness = −0.10). These large transient divergence movements would presumably have caused loss of binocular fusion, but we cannot be sure because we did not ask subjects to report fusion breaks if they occurred. In any case, owing to the method by which the PRL is determined (see Methods) these fixation losses will not cause systematic shifts or biases in the PRLs reported elsewhere in this article.

The eye motion traces also frequently exhibited a sawtooth waveform with a period equal to the frame duration. This is an artifact that can be attributed to torsional eye movements (Otero-Millan, Roberts, Lasker, Zee, & Kheradmand, 2015; Bowers, Boehm, & Roorda, 2019; Hofmann et al., 2022) and/or rotation of the head (and eyes) during the course of the measurement. The artifact arises because of constraints of the strip-based method to extract eye motion. Because the cross-correlation technique does not account for rotation of the strips, if the current frame is rotated relative to the reference frame, the best alignment can only be achieved by shearing the rotated image, which manifests as a sawtooth movement in the eye motion trace. The amplitude of the sawtooth indicates the amount of torsion or rotation, which for this system has a ratio of 5.95 minarc per degree. The amplitude of the sawtooth in Figure 5 indicates approximately a 0.5° rotation of the image. Importantly, the sawtooth artifact has minimal impact on the overall position of the eye trace for analysing the PRLs because it oscillates about the mean eye position. We note that a quantitative analysis of the sawtooth artifact could, in principle, be used to measure changes in torsion with vergence (Hofmann et al., 2022), but we could not control for or measure changes in head position, so we did not do so.

PRLs varied slightly from one condition to the next. The central tendencies and variation are shown in Figure 6, which shows how the position of the PRLs and ISOA in the two eyes varied across trials and conditions. The contours are plotted directly on the master retinal reference image for each eye of each subject. The PRLs are indicated by star symbols within each contour and represent the peak of the kernel density function used to determine the isoline contour for each condition. The variation from trial to trial was largely consistent with previous findings (Putnam et al., 2005; Bowers et al., 2021; Reiniger et al., 2021). Generally the non-dominant (left) eye tended to have larger isoline contour areas (average ISOA across all subjects and all binocular conditions = 83.18 minarc^2^), which means that fixation was less stable in that eye than in the dominant (right) eye (average ISOA = 54.11 minarc^2^), but this trend was not quite significant (repeated-measures analysis of variance (ANOVA), *F* (2, 3) = 6.98, *p* = 0.057). There were also more instances of statistically significant differences between PRLs in the non-dominant (left) than in the dominant (right) eye. This shift is analysed further in the next section.

To further investigate the changes in fixation disparity with increasing vergence demand, we generated histograms of the trial-by-trial horizontal eye positions. Figure 7 shows those positions for each subject and vergence demand. The upward histograms are for the left eye and the downward ones are for the right eye. Each histogram is positioned so that zero on the *x* axis represents the relative location of the peak of the kernel density function (i.e., the PRL) for the monocular condition for that subject. Data were only included in cases in which there were recordable traces from both eyes, which occurred in 40 of the 45 pairs of video in the binocular trials.

The two histograms are close to being aligned with one another, but the central tendencies are systematically shifted in the direction of an exo fixation disparity with increasing vergence demand. There are some other trends. The variance of the distribution is generally greater in the non-dominant (left) eye than in the dominant (right) eye and there were long tails in some of the distributions that were mostly due to brief divergent movements of the non-dominant (left) eye relative to the fixation target (as evident by the trend toward positive skewness in the left distribution).

The horizontal shifts in PRLs with increasing vergence demand are consistent with an increasing exo disparity as many others have observed (Hebbard, 1962; Ogle et al., 1967; Kertesz & Lee, 1987; Fogt & Jones, 1998a; Jaschinski, 2018). As we said earlier, we used the PRL obtained in the monocular condition to define zero disparity. By this definition, non-zero disparity represents horizontal shifts in PRLs in the binocular conditions relative to their locations in the monocular condition. Figure 8 plots the horizontal shifts in PRLs as a function of vergence demand; the left panel plots the shifts in the left eye and the right panel those in the right eye. The shifts were larger and more systematic in the non-dominant (left) eye than in the dominant (right) eye. With increasing vergence demand, the PRL tended to shift rightward on the left retina (fundus view), while staying relatively constant on the right retina. This is in agreement with the very definition of motor dominance where the non-dominant eye deviates as the motor-dominant eye maintains fixation (Crider, 1935; Coren, 1973). The left panel of Figure 9 shows that the left-eye shifts caused systematic changes in fixation disparity. That panel plots fixation disparity as a function of vergence demand for each of our five subjects. The negative slopes of these data are consistent with increasing exo disparity with increasing demand. This trend is substantiated by a significant main effect of vergence demand (repeated-measures ANOVA, *F* (2, 8) = 12.19, *p* < 0.01). The effect of vergence demand was also significant for the comparison between 0° and 4° (*p* < 0.05) and between 0° and 8° (*p* < 0.01). It did not reach significance for the comparison between 4° and 8° (*p* = 0.087) (post hoc Holm correction).

We next compare our findings with those of previous studies. Subjective and objective measurements were obtained from three previous reports: (Hebbard, 1962; Kertesz & Lee, 1987; Fogt & Jones, 1998a). Subjective measurements were also obtained from another previous report: (Ogle et al., 1967). Some of the studies stated the vergence demands in vergence angles while others stated them in prism diopters. To compare results across the various studies we needed to calculate the vergence demands in the same units. We used the following equations to do so:

(1) $$\mu =\tan^{-1}\left(\frac{i}{d}\right)$$

(2) $$\mu =\tan^{-1}\left(\frac{\Delta +50(\frac{i}{d})}{100}\right),$$

where μ is vergence demand in degrees, *i* is inter-ocular distance in meters, *d* is physical distance to the fixation target in meters, and Δ is prism diopters applied in front of an eye. If prisms are placed in front of both eyes, we use the clinical convention that their power is half the stated vergence demand. Note that these equations take into account the subject’s inter-ocular distance and the physical distance to the fixation target: two things that are not normally done in papers reporting fixation disparity owing to the introduction of prisms in front of the eyes.

In the right panel of Figure 9 we plot previous data for demands of −2 to +10° to enable comparison with our data. With the exception of the Fogt and Jones data, the previous results are quite consistent with ours. Because our method is not affected by potential artifacts described in the Introduction, we initially thought we would observe smaller fixation disparities than previous reports; we did not. So we conclude that PRLs shift by small amounts with increasing vergence demand to create a standing fixation disparity, even when measured objectively from the retinal images.

We also observed vertical shifts in PRLs in binocular relative to monocular viewing. Figure 10 plots those shifts for each of our five subjects. Vertical shift is plotted as a function of horizontal vergence demand. The left and center panels show the shifts in the left and right eyes, respectively. The right panel shows the vertical disparity created by those shifts. As you can see, the shifts were small and unsystematic. Specifically, there was no systematic change in vertical disparity caused by changes in vergence demand. Thus, unlike horizontal shifts in PRLs, vertical shifts were small and not dependent on the stimulus to horizontal vergence.

## Discussion

We investigated changes in fixation disparity as the vergence demand of the fixation target was changed. We recorded the exact position of the stimuli on the retinal surface, thereby bypassing the need to infer the location of a target on the retina from recordings taken from the eye’s anterior structures as was done in previous objective measures of fixation disparity.

The main result is that subjects showed an increase in static objective fixation disparity as the vergence demand of the target was increased. These offsets were small, but all subjects exhibited the same trend: increasing exo fixation disparity with increasing demand for convergence. As shown in Figure 9, these increases in disparity were comparable to what has been found in other studies relying on objective and subjective measures. It is important to note that the fixation disparities we observed were smaller than the amplitude of Panum’s fusional area (Schor & Tyler, 1981), so subjects should have been able to maintain a single fused percept even as the stimuli shifted on the retina with vergence demand.

With our experimental apparatus, it will eventually be possible to perform a subjective fixation-disparity test simultaneous with the imaging. Unfortunately, the apparatus does not currently have that capability, but we believe our results provide strong support for the notion that the non-zero fixation disparities usually observed with subjective methods are indeed caused by shifts in the PRL.

The fixation disparities were primarily caused by shifts in fixation of the non-dominant eye, which was the left eye in all of our subjects (Figure 8). This finding is consistent with previous investigations that explored several sensory and motor differences between dominant and non-dominant eyes (Li et al., 2010). In our study, the non-dominant eye exhibited systematic shifts in PRL with increasing vergence demand (Figure 6). It also exhibited greater variability (Figure 6), including more frequent deviations in fixation that were large enough to place the target outside of Panum’s fusional area presumably causing loss of binocular fusion (Figure 5). The more frequent deviations caused greater skewness in the histograms (Figure 7).

Our findings suggest an important difference in fixation loci under monocular and binocular viewing conditions. Recall that the PRL, measured monocularly, is quite stable across days (Kilpeläinen et al., 2021; Reiniger et al., 2021) and tasks (Bowers et al., 2021). But we observed systematic shifts in PRLs under binocular conditions with changes in vergence. The shifts we observed were large enough to have elicited corrective microsaccades under monocular conditions. For example, Poletti, Listorti, and Rucci (2013) found that an eye makes corrective microsaccades when the target falls just 5 minarc from the PRL. Ratnam (2017) reported that three of her six subjects (one who is subject 10003 in this study) would repeatedly make involuntary, corrective microsaccades to stimuli placed as little as 5 minarc away from the PRL. Despite this exquisite sensitivity to gaze shifts in monocular viewing, our subjects seemed to tolerate shifts in fixation loci in one eye as large as 8 minarc when they were viewing targets binocularly (Figure 9). Perhaps this is not too surprising because the requirements for maintaining high-fidelity monocular vision are different than those for binocular vision. To maintain a stable vergence posture, for example, it may be advantageous to converge slightly less than the demand in an effort to maintain stable closed-loop control (Schor, 1979) than to place the image at the monocular PRL. Furthermore, the signal to initiate a corrective eye movement in binocular viewing may be attained after binocular fusion. There are two kinds of corrective movements that could occur: vergence and version. There may be no need to change vergence unless diplopia (double vision) occurs and Panum’s fusion area reduces the probability of diplopia even when the eyes are not perfectly converged. There may be no need to change version (a leftward or rightward movement) unless a change in perceived visual direction occurs and the fact that the dominant eye is given more weight in that computation (Banks, Van Ee, & Backus, 1997; Sridhar & Bedell, 2011) provides some protection against a change in perceived direction. Further studies under binocular viewing conditions invoking more active discrimination tasks could evaluate how much PRL shift the visual system is ready to trade at the cost of poorer individual visual acuity (Westheimer, 1979b) and stereoacuity (Blakemore, 1970).

Theoretically, the two eyes need to have a specific location that they can treat as a 0,0 position to calculate binocular disparity accurately. If this position shifts on the retina under different binocular viewing conditions, the estimation of binocular disparity becomes more complicated. We know that area V1 is the first stage of disparity estimation, and that the estimate is in retinal coordinates. Said another way, V1 neurons signal the absolute disparity of the stimulus (Cumming & Parker, 1999). Vergence-related changes in the two eyes’ PRLs will affect the estimate of disparity at this stage of processing. But we believe that they will not affect perceived depth because the depth from disparity is based on relative disparity, which is the disparity between two or more objects (Westheimer, 1979a). Relative disparity is calculated by some neurons in primate area V2 (Thomas, Cumming, & Parker, 2002). The effect of changing vergence is canceled in the calculation of relative disparity because the eye movement equally affects the retinal images of both objects, and therefore the relative disparity is unaffected.

There is a considerable variation in experimental methodology between studies that have examined fixation disparity. For a thorough review of previous studies, and their methodologies and results see Fogt (2023). However, as mentioned, most studies of fixation disparity have manipulated vergence demand by placing prisms in front of the eyes, and fewer have manipulated demand by varying the distance to the binocular fixation target. Prisms affect how the eyes must converge to fuse the fixation target but they do not affect the distance to which the eyes should accommodate to sharpen the retinal image of the target. The prism method, therefore, creates a conflict between the stimulus to vergence and the stimulus to accommodation: the vergence-accommodation conflict (Wann, Rushton, & Mon-Williams, 1995; Hoffman, Girshick, Akeley, & Banks, 2008). For the eyes to converge to a different distance than they accommodate requires overcoming the neural linkage between the vergence and accommodative systems (Fincham & Walton, 1957). Our experiment has a similar constraint. When we altered vergence demand, we did so by rotating one of the arms of the apparatus and that did not affect the optical distance of the target which in turn did not affect the accommodative demand. Methods in which the actual target distance is changed are consistent with what occurs in natural viewing: that is, the distances to which the eyes must converge and accommodate are the same. It seems plausible therefore that fixation disparity will be smaller when the vergence and accommodation demands are the same, but we know of no studies that have directly compared results with and without vergence-accommodation conflict.

Our study concerned PRLs in normally sighted individuals. PRLs are of course studied in clinical populations as well, including patients with macular degeneration or strabismus. With macular degeneration the PRL is usually an eccentric region of the retina—typically just outside the central scotoma—to which patients direct fixated targets (Legge & Chung, 2016). With strabismus, patients direct binocularly fixated targets to eccentric parts of the retina in the deviating eye. Interestingly, these same patients usually direct monocularly fixated targets in the deviating eye to the fovea (Scheiman & Wick, 2008). Thus, PRLs in strabismics shift in the non-dominant eye much in the way we observed with our normally sighted subjects; but the shift is much larger in strabismics than we observed. The clinical observations and the ones we report here add nuance to the definition of the PRL. Its position depends on whether the subject is fixating monocularly or binocularly and on the vergence demand.

## Conclusions

We used a binocular scanning laser ophthalmoscope to study where on the retinas the target falls when people fixate a target binocularly. We observed small but systematic shifts in the PRLs, resulting in an exo fixation disparity (underconvergence) as the vergence demand of the stimuli was increased. The magnitude of the disparity was similar to other reports in the literature measured both objectively and subjectively. The fixation disparities were largely due to PRL shifts in the non-dominant eye.

## Supplementary Material

Supplement 1
